# Optimal Monitoring of Weekly IGF-I Levels During Growth Hormone Therapy With Once-Weekly Somapacitan

**DOI:** 10.1210/clinem/dgaa775

**Published:** 2020-12-12

**Authors:** Rasmus Juul Kildemoes, Michael Højby Rasmussen, Henrik Agersø, Rune Viig Overgaard

**Affiliations:** 1 Quantitative Clinical Pharmacology, Novo Nordisk A/S, Søborg, Denmark; 2 Global Development, Novo Nordisk A/S, Bagsværd, Denmark

**Keywords:** IGF-I, IGF-I SDS, long-acting growth hormone, monitoring, once-weekly dosing

## Abstract

**Context:**

Somapacitan is a long-acting growth hormone (GH) in development for once-weekly treatment of GH deficiency (GHD). Optimal monitoring of insulin-like growth factor-I (IGF-I) levels must account for weekly IGF-I fluctuations following somapacitan administration.

**Objective:**

To develop and assess the reliability of linear models for predicting mean and peak IGF-I levels from samples taken on different days after dosing.

**Design:**

A pharmacokinetic/pharmacodynamic model was used to simulate IGF-I data in adults and children following weekly somapacitan treatment of GHD.

**Setting and Patients:**

39 200 IGF-I profiles were simulated with reference to data from 26 adults and 23 children with GHD.

**Intervention(s):**

The simulated dose range was 0.02 to 0.12 mg/kg for adults and 0.02 to 0.16 mg/kg for children. Simulated data with >4 average standard deviation score were excluded.

**Main Outcome Measure(s):**

Linear models for predicting mean and peak IGF-I levels based on IGF-I samples from different days after somapacitan dose.

**Results:**

Robust linear relationships were found between IGF-I sampled on any day after somapacitan dose and the weekly mean (R^2^ > 0.94) and peak (R^2^ > 0.84). Prediction uncertainties were generally low when predicting mean from samples taken on any day (residual standard deviation [RSD] ≤ 0.36) and peak from samples taken on day 1 to 4 (RSD ≤ 0.34). IGF-I monitoring on day 4 and day 2 after dose provided the most accurate estimate of IGF-I mean (RSD < 0.2) and peak (RSD < 0.1), respectively.

**Conclusions:**

Linear models provided a simple and reliable tool to aid optimal monitoring of IGF-I by predicting mean and peak IGF-I levels based on an IGF-I sample following dosing of somapacitan. A short visual summary of our work is available (1).

Growth hormone (GH) therapy has a well-established safety and efficacy profile for the treatment of GH deficiency (GHD) in children (2) and adults (3). The efficacy of GH therapy is dependent upon making the correct diagnosis, administering the appropriate dosage of GH, and maintaining patient adherence and persistence with treatment (4, 5). Treatment for GHD in both pediatric and adult patients is by daily subcutaneous injections, typically administered until the child reaches adult height, or lifelong in the case of adult GHD (6, 7). However, the necessity of daily injections is associated with noncompliance and increased healthcare costs (8, 9). Depending on the methods used, estimates of the prevalence of nonadherence range from 5% to 82% in children (10) and around 48% in adults (11).

Challenges to adherence and persistence with daily GH therapy include device limitations, inconvenience of required dose frequency, and lack of perceived benefit (8, 10-15). Furthermore, adherence has been reported to worsen over time, potentially affecting long-term outcomes during once-daily GH treatment (16).

Since adherence to daily injections of GH has not been optimal, it has been postulated that, by facilitating increased adherence, a long-acting GH formulation that could be given weekly, biweekly, or even more infrequently might lead to better clinical outcomes (17). Data from a questionnaire study involving adult patients who had participated in clinical trials with long-acting GH indicated that patients preferred weekly over daily injections, especially with regard to the lower number of injections and lower time expenditure for injections (13).

Once-weekly somapacitan (Novo Nordisk A/S, Bagsværd, Denmark) is a novel, reversible, albumin-binding GH derivative in clinical development, with a small albumin-binding moiety (1.2kDa) attached to the GH molecule. This facilitates reversible binding to circulating endogenous albumin, reducing clearance and extending half-life to allow once-weekly administration (18, 19). Evidence supports that once-weekly subcutaneous administration of somapacitan in adults and children is well tolerated, with a good safety profile (20-23).

Insulin-like growth factor (IGF-I) is an indicator for bioavailable GH and a biomarker for GH response (24). In children, IGF-I is monitored to ensure adherence and long-term safety of GH treatment (24, 25). In adults, GH treatment regimens use individualized dose-titration strategies targeting normalization of serum IGF-I to account for inter-individual differences in GH sensitivity such as age, sex, body mass index (BMI), and various other patient characteristics (26, 27). The use of individualized, stepwise dose adjustments based on serum IGF-I levels has resulted in improved treatment efficacy, provided the patient is treatment-adherent, and reductions in reported adverse events (28, 29). Reliable monitoring of IGF-I levels is, therefore, essential for ensuring the correct GH dose during treatment of GHD, as well as for safety assessments. In clinical practice, IGF-I levels are converted to standard deviation score (SDS) to allow standardization between individuals with a range of baseline levels and facilitate clinical decisions (30, 31). According to international guideline recommendations for adults with GHD, the dose of GH replacement should be adjusted based on clinical efficacy, side effects, and serum IGF-I SDS, with the aim of maintaining IGF-I SDS within the upper normal range (3, 32).

The correct assessment of weekly IGF-I levels during treatment with any long-acting GH needs to consider fluctuations in IGF-I over the dosing interval (33). During treatment with daily GH, IGF-I levels increase and decrease over the daily dosing interval, with relatively small fluctuations. In comparison, the IGF-I profile following any long-acting GH administered weekly will fluctuate over the dosing interval with larger fluctuations (as illustrated in Fig. 1). As a consequence, monitoring of IGF-I levels following long-acting GH products must take into account the time after dose that the IGF-I sample has been collected. For somapacitan, IGF-I fluctuation over a dosing interval is well characterized in clinical trials in adults and children with GHD and is quantified using population pharmacokinetic (PK)/pharmacodynamic (PD) modeling (34).

**Figure 1.**
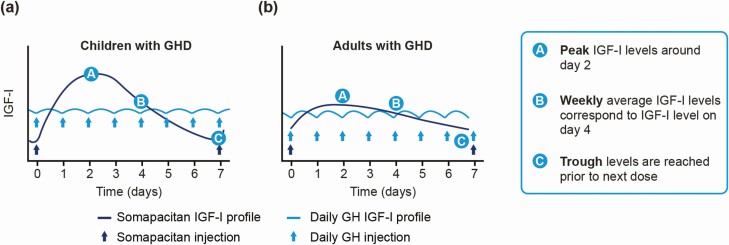
Modeled IGF-I SDS fluctuations over a week demonstrate peak, mean and trough IGF-I levels of weekly somapacitan vs IGF-I levels following daily GH injections in a) children and b) adults. Abbreviations: GH, growth hormone; GHD, growth hormone deficiency; IGF-I, insulin-like growth factor-I.

This study aimed to describe the relationship between IGF-I levels monitored on any day post-somapacitan dosing, and to evaluate whether reliable estimates of weekly mean and peak IGF-I can be obtained from a single IGF-I sample taken during weekly dosing with somapacitan. The objective was to develop and assess the reliability of a tool to predict the mean and peak of weekly IGF-I profiles by IGF-I monitoring during treatment with somapacitan.

## Materials and Methods

### Population PK/PD model and data

A PK/PD model was available for simulation of full PK and IGF-I profiles following somapacitan dosing. The model has previously been qualified and published (34). Data for the PK/PD model were derived from 3 phase 1 trials of somapacitan, including data from healthy adults (ClinicalTrials.gov identifier NCT01514500; first human dose trial) (35) and from 2 randomized studies of patients with GHD. The first involved adults (ClinicalTrials.gov identifier NCT01706783) (22) and the second involved children (ClinicalTrials.gov identifier NCT01973244) (21).

### Analysis of IGF-I

The available PK/PD model was based on serum concentrations of IGF-I analyzed at analytical laboratory Laboratorium für Klinische Forschung GmbH, Schwentinental, Germany using commercially available assay kits (Immuno Diagnostic Systems immunoassay [IDS-iSYS assay]). Normal reference ranges for the calculation of SDS were based on the publication by Bidlingmaier et al (30). It was not the scope of this analysis to assess the impact of assay variability on IGF-I calculation.

### Simulation

Data for the current analysis were generated by full parametric simulation of a population of adults with GHD and children with GHD. The demographic characteristics of subjects were retained as in the data that were used to develop the PK/PD model and all subjects were simulated at all dose levels investigated in the trials. Thus, all adults with GHD (n = 26) and children with GHD (n = 23) were simulated at 4 dose levels (0.02-0.12 mg/kg for adults and 0.02-0.16 mg/kg for children), and simulations were repeated 200 times to obtain a population of 39 200 new simulated IGF-I profiles (34). Steady-state IGF-I profiles were constructed from individual predictions using a 4-hour grid from 0 to 168 hours after dose. Simulations were performed using nonlinear mixed effects modeling software (NONMEM).

**Figure 2.**
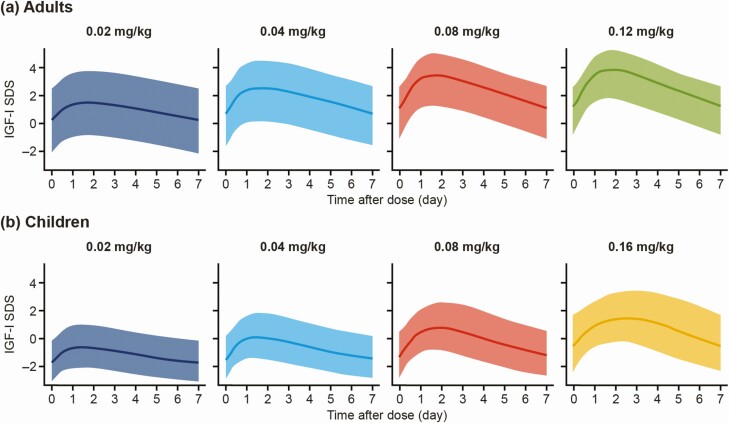
Simulated data used as the basis for linearization: individual predicted IGF-I SDS profiles for a) adults and b) children by time (in days) after dose and somapacitan dose. Profiles with an average IGF-I SDS > 4 over a week were excluded. Lines represent mean values. Shaded areas represent 5th to 95th percentiles. Abbreviations: IGF-I, insulin-like growth factor-I; SDS, standard deviation score.

To ensure optimal predictions in the clinically relevant range for adults with GHD, and to avoid bias, simulated profiles with an average IGF-I SDS > 4 over a week were not included in the analysis. However, profiles with a peak IGF-I SDS > 4, but average IGF-I SDS < 4, were not excluded in order to maintain the clinical relevance of the IGF-I profiles. This exclusion concerned 30.8% of simulated profiles in adults, primarily from the higher dose levels (0.08 and 0.12 mg/kg), reflecting that only a subset of adults need the higher dose levels to obtain IGF-I response. A graphical summary of the IGF-I SDS data after exclusion of these data is seen in Fig. 2.

### Linearization

For each potential IGF-I sample time after dose (TAD), linear models were used to fit the correlation between individual predicted IGF-I SDS values and the mean and peak across a week at steady state.

Covariates were included sequentially based on inspection of trends in the residuals after model fit. Dose, body weight, age, and sex were considered for inclusion.

For adults with GHD, the best fit to data included a dose (in mg) covariate with body weight as an interaction. For each TAD, the following equation applied in adults with GHD (Y_AGHD_), given the intercept (Int), slope of IGF-I SDS (Slp.SDS), IGF-I SDS (SDS), slope of dose (Slp.Dose), somapacitan dose (Dose), slope of interaction between dose and body weight normalized to 80 kg (Slp.Dose_BW80_), and body weight (BW):


$$\begin{array}{ll} Y_{AGHD}= & \;\;Int+Slp.SDS\cdot SDS+Slp.Dose\cdot Dose\\ & +Slp.Dose_{BW80}\cdot Dose\cdot (BW-80kg) \end{array}$$

For children, the best fit to data included a logarithmic dose level (in mg/kg) covariate (slope of log dose [Slp.logDose]), but no further covariates (including body weight) were indicated. For each TAD, the following equation applied in children with GHD (Y_GHD_):


$$\begin{array}{l} Y_{GHD}=\;Int+Slp.SDS\cdot SDS+Slp.logDose\cdot \ln\left(\frac{Dose}{0.16mg/kg}\right) \end{array}$$

The degree of linearization was evaluated on the correlation coefficient R^2^. An R^2^ value ≥0.9 indicates a tight linear relationship.

The precision of weekly mean and peak predictions was evaluated using the residual standard deviation (RSD) and the 90% prediction interval was calculated as ±1.645*RSD. Linear models were fitted in R version 3.5.3.

In addition to prediction of weekly mean and peak, prediction of IGF-I levels on any other day was performed using the same approach in order to illustrate the profiles.

## Results

### Simulations

Fig. 2 shows simulations in adults and children used for analysis (not including profiles with mean IGF-I SDS > 4 over a week). As can be seen, the profiles included IGF-I SDS levels in the clinically relevant range (across and slightly above the normal IGF-I SDS range of –2 to +2).

### Models

The parameters for the models at each day after dose are provided in Table 1. Note that values on day 0 and day 7 are identical because it was assumed that dosing was performed at steady state.

**Table 1.** Parameters for Prediction of Mean IGF-I SDS (a, b) and Peak IGF-I SDS (c, d) in Adults With GHD and Children With GHD, Based on an IGF-I Sample Taken on Days 0–7 After Somapacitan Dose

*a) Adults with GHD—mean IGF-I SDS*							
Day	Int	Slp.SDS	Slp.Dose	Slp.Dose_BW80	RSD	R^2^	PI 90% ±SDS
0	0.681	0.965	0.105	-0.00176	0.36	0.94	0.6
1	-0.282	0.959	-0.0400	0.000842	0.29	0.96	0.5
2	-0.334	0.936	-0.0585	0.00144	0.26	0.97	0.4
3	-0.242	0.947	-0.0317	0.000850	0.19	0.98	0.3
4	-0.0495	0.968	-0.000638	0.0000950	0.11	0.99	0.2
5	0.196	0.981	0.0330	-0.000625	0.14	0.99	0.2
6	0.450	0.980	0.0690	-0.00126	0.25	0.97	0.4
7	0.681	0.965	0.105	-0.00176	0.36	0.94	0.6
***b) Children with GHD—mean IGF-I SDS***							
**Day**	**Int**	**Slp.SDS**	**Slp.logDose**	**RSD**	**R** ^**2**^		**PI 90% ±SDS**
0	1.24	0.950	0.353	0.29	0.94		0.5
1	-0.350	1.02	0.0990	0.28	0.95		0.5
2	-0.738	0.961	-0.0959	0.27	0.95		0.4
3	-0.609	0.936	-0.162	0.20	0.97		0.3
4	-0.208	0.917	-0.0926	0.16	0.98		0.3
5	0.293	0.912	0.0510	0.19	0.98		0.3
6	0.790	0.927	0.207	0.23	0.96		0.4
7	1.24	0.950	0.353	0.29	0.94		0.5
***c) Adults with GHD—peak IGF-I SDS***							
**Day**	**Int**	**Slp.SDS**	**Slp.Dose**	**Slp.Dose_BW80**	**RSD**	**R** ^**2**^	**PI 90% ±SDS**
0	1.11	0.919	0.200	-0.00337	0.64	0.84	1.1
1	0.0742	1.02	0.0319	-0.000782	0.19	0.98	0.3
2	0.0149	0.997	0.0114	-0.000145	0.091	1.0	0.1
3	0.139	0.986	0.0462	-0.000795	0.22	0.98	0.4
4	0.362	0.982	0.0845	-0.00159	0.33	0.96	0.5
5	0.626	0.972	0.123	-0.00231	0.44	0.92	0.7
6	0.886	0.950	0.162	-0.00291	0.54	0.88	0.9
7	1.11	0.919	0.200	-0.00337	0.64	0.84	1.1
***d) Children with GHD—peak IGF-I SDS***							
**Day**	**Int**	**Slp.SDS**	**Slp.logDose**	**RSD**	**R** ^**2**^		**PI 90% ±SDS**
0	2.10	0.866	0.562	0.51	0.84		0.8
1	0.534	1.05	0.239	0.18	0.98		0.3
2	0.118	1.00	0.0271	0.092	1.0		0.2
3	0.295	0.945	-0.00764	0.20	0.98		0.3
4	0.737	0.887	0.103	0.34	0.93		0.6
5	1.23	0.852	0.269	0.42	0.89		0.7
6	1.70	0.850	0.426	0.48	0.86		0.8
7	2.10	0.866	0.562	0.51	0.84		0.8

Abbreviations: GHD, growth hormone deficiency; IGF-I, insulin-like growth factor-I; int, intercept; PI, prediction interval; R^2^, correlation coefficient; RSD, residual standard deviation; SDS, standard deviation score; slp.SDS, slope of insulin-like growth factor-I standard deviation score; slp.Dose, slope of dose; slp.logDose, slope of log dose; slp.Dose_BW80, slope of interaction between dose and body weight (normalized to 80 kg).

**Figure 3.**
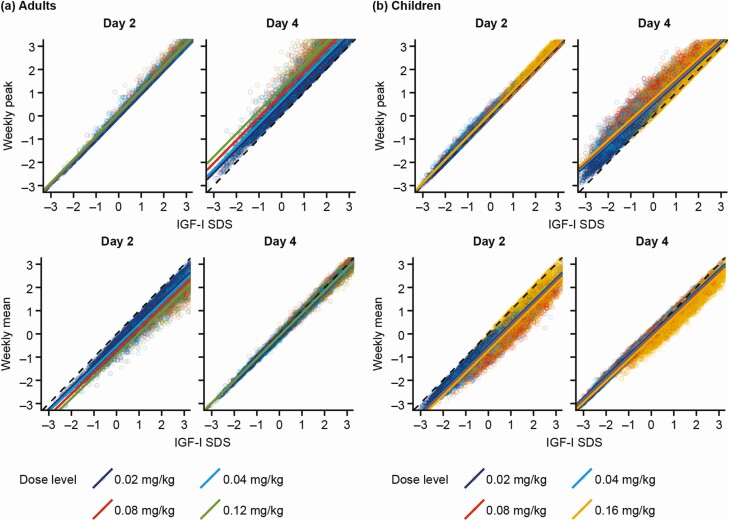
Correlations between individual predicted IGF-I SDS on days 2 and 4 after somapacitan dosing versus the weekly peak (top panel) and the weekly mean (bottom panel) IGF-I SDS levels for a) adults with GHD and b) children with GHD. The line of identity is shown (in black, dashed line at 45°). Abbreviations: AGHD, adults with growth hormone deficiency; GHD, growth hormone deficiency; IGF-I, insulin-like growth factor-I; SDS, standard deviation score.

A tight linear relationship between IGF-I SDS on any day and the weekly mean across the full dose ranges was observed and these were well described by the models (Fig. 3).

**Figure 4.**
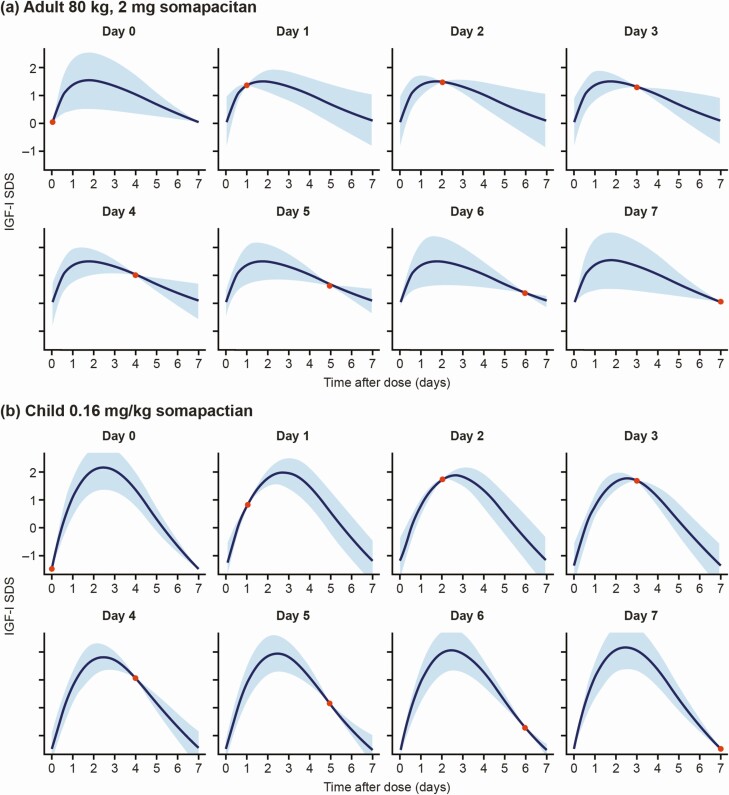
Illustration of the predicted IGF-I SDS profiles and the 90% prediction interval for a sample taken on days 0–7 after somapacitan dosing for a typical a) adult with GHD (2 mg dose of somapacitan) and b) child with GHD (0.16 mg/kg somapacitan). The solid line and shaded area represent the predicted IGF-I SDS profile with a 90% prediction interval based on a single sample (red dot). Abbreviations: GHD, growth hormone deficiency; IGF-I, insulin-like growth factor-I; SDS, standard deviation score.

**Figure 5.**
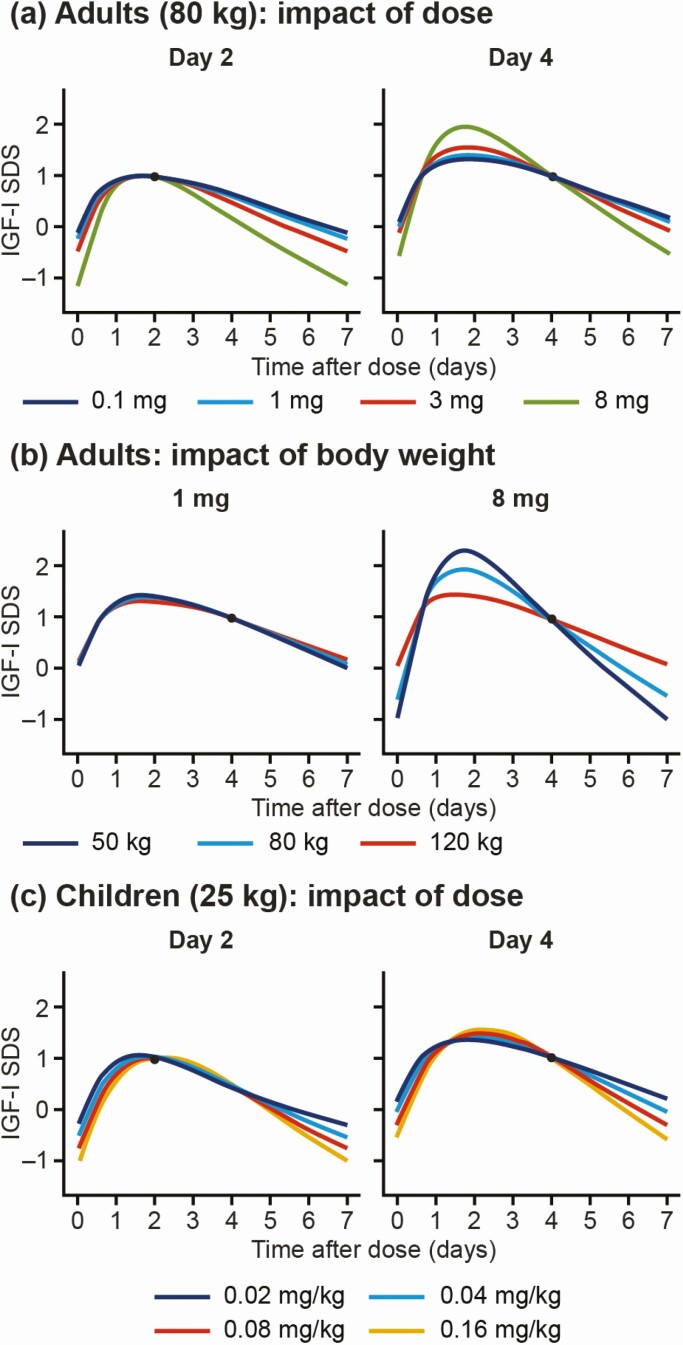
Illustration of the impact of somapacitan dose in a) adults (0.1, 1.0, 3.0, and 8 mg) and c) children (0.02, 0.04, 0.08, and 0.16 mg/kg), and of b) body weight in adults (50, 80, and 120 kg) on predicted IGF-I SDS profiles, for a single IGF-I sample. The blue dot indicates the single IGF-I sample in each case. Abbreviations: IGF-I, insulin-like growth factor-I; SDS, standard deviation score.

For illustration, the expected weekly IGF-I SDS profile and the uncertainty based on a single measurement were constructed using the linear relationships between a sample taken on different days and the most likely IGF-I SDS levels on any day over the entire weekly profile. The predicted profiles, with the uncertainties associated with sampling on different days after dose, are illustrated in Fig. 4. The effects of dose and body weight on the profile are illustrated in Fig. 5. For both adults and children, increased doses were associated with larger fluctuations in IGF-I from peak to trough, whereas flatter curves were estimated at lower dose levels. In adults, low body weight was also associated with larger peak-to-trough fluctuations; this association was not apparent in children when accounting for the dose in mg/kg.

Extended tables with parameters for prediction of mean and peak IGF-I SDS every 4 hours for 7 days after dose can be viewed in Tables S1 to S4 in the Supplementary Material (36).

### Evaluation of predictive performance

Tight linear relationships were demonstrated between IGF-I SDS on any day and mean IGF-I SDS during the week, as well as between IGF-I SDS on days 1 to 5 and peak value (Table 1). In both children and adults, the strongest correlations were observed between the day 4 sample and mean, as well as between the day 2 sample and peak value.

Overall, weekly mean IGF-I SDS could be predicted with good precision given an IGF-I sample on any day in both children and adults (RSD < 0.4 for adults and RSD < 0.3 for children) (Table 1a and 1b). The most accurate prediction of the mean IGF-I SDS value was obtained when sampling was done on day 4, where RSD < 0.2 in both adults and children (90% prediction interval ±0.2 and ±0.3 SDS, respectively) (Table 1a and 1b).

The weekly peak IGF-I SDS value was best predicted with an IGF-I sample obtained on days 1 to 4 in both children and adults (RSD < 0.4) (Table 1c and 1d). The most accurate prediction of the peak IGF-I SDS value was obtained when sampling on day 2, where RSD < 0.1 in adults and children (90% prediction interval ±0.1 and ±0.2 SDS, respectively) (Table 1c and 1d).

## Discussion

Using simulated data from a PK/PD model developed from 3 phase 1 trials, we produced linear models that provide simple tools to predict mean and peak IGF-I SDS levels in children and adults with GHD based on an accurate IGF-I sample following somapacitan dosing at steady state. The model showed that IGF-I measurements taken on any day after the dosing day can be used to predict mean and peak IGF-I SDS levels during the dosing period. Overall, mean IGF-I SDS was best predicted by a single sample obtained 4 days after dosing, whereas a sample taken on day 2 after dosing provided the best estimate of peak IGF-I SDS levels in children and adults.

To our knowledge, model-based interpretation of IGF-I SDS during monitoring will be relevant for any long-acting GH derivatives. One such model was published for MOD-4023, a long-acting human GH based on carboxy-terminal peptide technology (37). The authors reported that mean IGF-I SDS was best predicted by IGF-I samples collected on day 4 after dose administration (33).

A reliable assessment of IGF-I SDS is important for dose titration and monitoring of adherence and safety. The mean IGF-I SDS is particularly important, because it reflects the overall systemic exposure to IGF-I and, as such, is generally regarded as a biomarker for the effects of exogenous GH. In contrast, peak IGF-SDS indicates the peak of the transient elevation that is expected and characteristic for a weekly GH product.

In adults, once maintenance doses are achieved, IGF-I should be measured regularly, such as every 6 to 12 months, to ensure that the level falls within the age-adjusted reference range (SDS between −2 and +2) (3). In children, the goal is to maintain serum levels of IGF-I SDS within the normal age-appropriate range for the majority of the treatment period (16). IGF-I SDS levels maintained within the normal range provide assurance that the GH dose is adequate and may be used to indicate compliance (6, 16). Abnormally high IGF-I SDS levels may be an indication that a reduction in GH dose is needed (16), whereas, in contrast, low levels of IGF-I SDS, especially if they are found in association with a low growth response, may indicate that an increase in dose is required (38). Clinical opinions may differ on the usefulness of peak, mean, and trough IGF-I SDS levels as tools to monitor and guide therapy (16). No evidence exists that transient elevations in IGF-I levels (above +2 SDS) are associated with GH-treatment-related safety issues in children (39, 40). The long-term risks of IGF-I levels outside of the normal range, of short or long duration, are not resolved (2).

Unlike the experience with daily GH, the timing of blood sampling in patients treated with long-acting GH must be considered, because IGF-I levels will fluctuate over a week. The PK and PD of long-acting GH preparations differ from that of daily GH; they also vary among different long-acting GH preparations. This variability needs to be taken into account to gauge the optimal timing of IGF-I measurements for each product (16). During the dosing interval of a long-acting GH, IGF-I will increase and then decrease over a period longer than 24 hours. The strong linear relationships documented in this work provide confidence that samples obtained at different times following somapacitan dose administration can be used to estimate mean and peak IGF-I SDS and may be useful in optimal monitoring of IGF-I levels. This may allow for flexibility and convenience for patients and physicians when choosing an appropriate sampling time after dose. However, it should be noted that this is an exploratory model only and is not approved for use.

Despite the underlying nonlinear PK/PD of somapacitan, the linearized models for IGF-I samples taken at various times after dose provided a reliable prediction of the weekly mean and/or peak IGF-I SDS, provided that the IGF-I sample is taken at steady-state dosing and is accurate. It is, therefore, a key assumption of the model that the IGF-I sample is taken during steady state, that is, where it can be assumed that the pre-dose levels are the same at the start and end of the dosing interval. If steady state has not been achieved at the time of sampling, this is not accounted for within the linear model, and subsequent predictions of IGF-I values may be biased. This is also the case if the somapacitan dose is changed. For precise prediction of mean and/or peak IGF-I SDS following a dose change, at least one dosing interval at the new dose must be allowed to pass before sampling, to ensure that steady state has been achieved (34).

A potential limitation of our model is that it was developed based on retrospective data. The model could be further validated and refined using prospective data. Another limitation of the predictive power of the model is that an accurate sample of IGF-I is required. Analysis results may vary from one sample to another due to technical reasons, such as assay variability, pharmacological reasons such as dose-to-dose variation in bioavailability, and temporal variations in physiology. This challenge, however, also applies to IGF-I monitoring of all weekly and daily GH products, and the most reliable way to limit the impact of variability is to monitor on a regular basis (2, 3).

## Conclusions

The linear model provided a simple and reliable tool to predict mean and peak IGF-I SDS levels over the week based on an accurate IGF-I sample following somapacitan dosing at steady state in adults and children with GHD. Mean IGF-I SDS was generally well predicted by IGF-I monitoring on any day and best predicted by IGF-I monitoring on day 4 after dose. Peak IGF-I was accurately predicted by IGF-I monitoring on day 1 to 4 after dose and best predicted by IGF-I monitoring on day 2.

## Data Availability

The simulated data generated for the analysis are not publicly available but can be made available upon reasonable request.

## Abbreviations

GH: growth hormone
GHD: growth hormone deficiency
IGF-I: insulin-like growth factor-I
PD: pharmacodynamic
PK: pharmacokinetic
RSD: residual standard deviation
SDS: standard deviation score
TAD: time after dose

## Additional Information

***Disclosure Summary:*** All authors are employees of Novo Nordisk A/S and own stocks or shares in the company.
